# Influence of Black and Brown Pigment Stone in Cholecystectomized Patients With Acute Biliary Pancreatitis

**DOI:** 10.4021/gr482w

**Published:** 2012-09-20

**Authors:** Shou-Wu Lee, Chi-Sen Chang, Han-Chung Lien, Teng-Yu Lee, Hong-Zen Yeh, Chun-Fang Tung

**Affiliations:** a Department of Internal Medicine, Division of Gastroenterology and Hepatology, Taichung Veterans General Hospital, Taichung, Taiwan; b Department of Medicine, Chung Shan Medical University, Taichung, Taiwan; c Department of Internal Medicine, Yang-Ming University School of Medicine, Taipei, Taiwan

**Keywords:** Bile duct stones, Cholecystectomy, Pancreatitis

## Abstract

**Background:**

Biliary tract stones account for the majority of cases with acute pancreatitis, and include black and brown pigment stones. The aim of the study was to compare the presentation and outcome in cholecystectomized patients with acute biliary pancreatits caused by black and brown pigment stones.

**Method:**

Data from patients with prior cholecystectomy and acute biliary pancreatitis were collected from January 2009 to August 2011. These cases were assigned to black or brown pigment stone groups according to the stone pattern. The general data, laboratory data, image findings and outcomes of the two groups were collected and analyzed.

**Results:**

A total of 98 enrolled patients, with 30 (30.6%) and 68 cases (69.4%) assigned to the black and brown pigment stone groups, respectively. The cases with black pigment stone had higher CT Severity Index scores, bilirubin, ALP, ALT, rates of cholangitis, and positive blood culture. In those with brown pigment stone, there was a higher number of ERCP evaluations performed.

**Conclusion:**

Cholecystectomized cases with biliary pancreatitis due to black pigment stone had a higher prevalence of laboratory cholestasis and a higher rate of cholangitis.

## Introduction

Acute pancreatitis has an incidence of between 4.8 to 24.2 cases per 100,000 and an associated mortality rate of up to 10% [1]. There is general acceptance that a diagnosis of acute pancreatitis requires two of the following three features: (1) abdominal pain characteristic of acute pancreatitis; (2) serum amylase and/or lipase over 3 times the upper limit of normal; and (3) characteristic findings of acute pancreatitis on computed tomography (CT) scan [2]. Alcohol and biliary tract stones together account for 80% of all cases, with stones alone causing 34% to 54% of cases [3], although there is some geographic variation. There are two generally accepted assessment scales used to evaluate the severity of pancreatitis attacks: Ranson’s criteria and the CT Severity Index [4, 5]. Bile duct stones include black pigment stones, migrated from gallstones, and brown pigment stones, formed primarily in the bile duct. However, little is known about recurrent biliary acute pancreatitis after cholecystectomy. The aim of the study was to compare the presentation and outcome of cholecystectomized patients with acute biliary pancreatits caused by black or brown pigment stones.

## Methods

Data from consecutive patients with a history of prior cholecystectomy, and acute pancreatitis, diagnosed by clinical, laboratory, and radiographic parameters, in our hospital, were retrospectively collected from January 2009 to August 2011. The etiology of acute pancreatitis was determined by a careful history, physical examination, and radiographic and laboratory findings. A biliary cause of acute pancreatitis was assumed with laboratory findings of cholestasis and results of imaging studies. All cases with acute biliary pancreatitis underwent abdominal CT examination and endoscopic retrograde cholangiopancreatography (ERCP) within 48 hours after acute pancreatitis occurred, and those with common bile duct (CBD) stones were enrolled into the study. Furthermore, these patients were assigned to two groups of black and brown pigment stone according to the stone color and pattern.

The general data of enrolled patients, including age, gender, disease severity, laboratory data, CT and ERCP image findings were recorded. All cases accepted intravenous hydration, parenteral analgesia, adequate nutritional support, and antibiotics if cholangitis or other infection occurred. The diagnosis of acute cholangitis comprised Charcot's triad of fever, jaundice, and right upper quadrant abdominal pain. The CT Severity Index of each case was evaluated in the initial admission, and Ranson’s scores were calculated in the initial and 48 hours after admission. The outcomes of each group, including combined cholangitis, positive blood or bile culture, one-month mortality, and hospital days were also collected and analyzed.

Data are expressed as standard deviation of mean for each of the measured parameters, or as a percentage of the total patient number. A P value below 0.05 was considered statistically significant. Pearson’s Chi-square test was used to compare gender, Ranson's scores, CBD stone numbers, juxtapapillary diverticula, rate of cholangitis, rate of positive bile culture. Fisher’s exact test was used to analyze CT severity index, number of ERCP procedures, as well as rates of positive blood culture and mortality. Independent t-test was used to compare age, C-reactive protein, bilirubin, alkaline phosphatase (ALP), alanine aminotransferase (ALT), CBD diameter, maximal CBD stone size, and hospital days.

## Results

A total of 98 consecutive patients diagnosed with biliary pancreatitis with bile duct stone documented by ERCP were enrolled in our study between January 2009 to August 2011. Among these patients, 30 (30.6%) and 68 cases (69.4%) were assigned to the black pigment stone group and brown pigment stone group, respectively. As shown in Table 1, there were no differences in age and gender ratio between the black and the brown pigment stone groups. Although the Ranson’s criteria scores of the two groups were similar, the CT Severity Index scores of the black pigment stone group were significantly higher than those of the brown pigment stone group (7 - 10 scores, 26.7% vs. 11.8%, P = 0.001).

**Table 1:** Demographic Data and Laboratory Results

	Black stone group (n = 30)			Brown stone group (n = 68)			P-value
	M ± SD	N	%	M ± SD	N	%	
Age	64.87 ± 13.99			71.35 ± 17.46			0.076^a^
Gender							0.698^b^
Male		20	(66.7%)		48	(70.6%)	
Female		10	(33.3%)		20	(29.4%)	
Severity							
Ranson’s scores							
≤ 2		20	(66.7%)		44	(64.7%)	0.851^b^
≥ 3		10	(33.3%)		24	(35.3%)	
CT severity index							
0-3		0			44	(64.7%)	0.001^c^
4-6		22	(73.3%)		16	(23.5%)	
7-10		8	(26.7%)		8	(11.8%)	
Laboratory data							
C-reactive protein	6.84 ± 6.52			4.20 ± 4.07			0.054^a^
Bilirubin	4.63 ± 1.30			2.50 ± 1.23			0.001^a^
ALP	405.87 ± 217.25			273.53 ± 222.68			0.008^a^
ALT	408.87 ± 335.28			244.41 ± 168.19			0.015^a^
Images							
CBD diameter	0.99 ± 0.24			1.01 ± 0.34			0.778^a^
CBD stone numbers							
1 - 2		20	(66.7%)		42	(61.8%)	0.641^b^
≥ 3		10	(33.3%)		26	(38.2%)	
Maximal CBD stone size	0.83 ± 0.30			0.85 ± 0.31			0.819^a^
Juxtapapillary diverticula		8	(26.7%)		28	(41.2%)	0.170^b^

^a^Independent T test; ^b^Pearson’s Chi-square test; ^c^Fisher’s exact test. Abbreviations: ALP, alkaline phosphatase; ALT, alanine aminotransferase; CBD, common bile duct; M ± SD, mean ± standard derivation; N, number of patients.

The laboratory data revealed that the black pigment stone group had significantly higher serum bilirubin, ALP and ALT than those of the brown pigment stone group (mean bilirubin 4.63 vs. 2.50, P = 0.001; ALP 405.87 vs. 273.53, P = 0.008; ALT 408.87 vs. 244.41, P = 0.015). There was also higher serum C-reactive protein in the black pigment stone group compared with that in the brown pigment stone group (mean 6.84 vs. 4.20, P = 0.054), although the differences were non-significant.

The imaging findings between the two groups are also summarized in Table 1. The CBD diameter, numbers of stones, and maximal stone size of these patients were similar. The ratio of juxtapapillary diverticula were non-significantly increased in the brown pigment stone group (41.2% in the brown pigment stone group, 26.7% in the black pigment stone group, P = 0.170).

The outcomes of the two groups are listed in Table 2. Only one ERCP procedure was required to achieve complete stone extraction in all patients with black stones, but a few cases (29.4%) with brown pigment stones needed ERCP more than once to remove all stones. The rates of cholangitis and positive blood culture were significantly increased in the black pigment stone group compared with those of the brown pigment stone group (cholangitis, 53.3% vs. 26.5%, P = 0.010; positive blood culture, 40% vs. 0%, P = 0.001). The two groups had similar percentage of positive bile culture (66.6% vs. 76.5%, P = 0.311) and hospital days (mean 13.27 vs. 12.94, P = 0.825). In addition, the mortality rate was non-significantly higher in the black pigment stone group (13.3% vs. 5.9%, P = 0.214) than in the brown pigment stone group.

**Table 2:** Clinical Characteristics

	Black stone group (n = 30)			Brown stone group (n = 68)			P-value
	M ± SD	N	%	M ± SD	N	%	
ERCP perform ≥ 2 times		0			20	(29.4%)	0.001^c^
Combined cholangitis		16	(53.3%)		18	(26.5%)	0.010^b^
Positive blood culture		12	(40.0%)		0		0.001^c^
Positive bile culture		20	(66.7%)		52	(76.5%)	0.311^b^
Mortality		4	(13.3%)		4	(5.9%)	0.214^c^
Hospital days	13.27 ± 5.06			12.94 ± 7.32			0.825^a^

^a^Independent T test; ^b^Pearson’s Chi-square test; ^c^Fisher’s exact test. Abbreviations: ERCP, endoscopic retrograde cholangiopancreatography; M ± SD, mean ± standard derivation; N, number of patients.

## Discussion

Acute biliary pancreatitis is initiated by obstruction of the ampulla of vater by migratory gallstones or by CBD stones. Persistent ampullary obstruction may be responsible for the progression from pancreatic edema to hemorrhage and necrosis. The first study to clearly establish the link between stones and acute pancreatitis was reported by Opie in 1901 [6]. In epidemiologic studies, biliary pancreatitis has been shown to be more common in women than in men and tends to occur more frequently in older age groups than in patients with pancreatitis due to alcohol ingestion [7]. In clinical practice, it is well known that biliary pancreatitis tends to follow an acute intermittent disease pattern, with individual attacks being clinically similar to those of other etiologies. Most cases of are mild and self-limited, requiring intravenous hydration, parenteral analgesia, and enteral or parenteral nutritional support, but bacteremia and ascending cholangitis are seen more commonly than biliary pancreatitis with other, nonobstructive causes [8]. Some studies have also shown a higher mortality rate among patients with biliary pancreatitis when compared to other causes of pancreatitis [9, 10]. Persistent CBD stones are treated by decompression of the ducts and removal of the stones either through surgical techniques, such as laparoscopic bile duct exploration, or through ERCP [11, 12].

Patients with a biliary etiology should undergo cholecystectomy in order to reduce the risk of recurrent acute pancreatitis or other biliary complications [13]. However, acute biliary pancreatitis may still occur after cholecystectomy. Sungler et al., in a series of 155 patients with acute biliary pancreatitis, found 22 (14%) with a history of prior cholecystectomy [14]. Similarly, Gloor B. reported 7 (13%) of 132 cases with acute biliary pancreatitis had a history of cholecystectomy [15]. In these patients, the possible etiologies include residual or recurrent bile duct stones, cystic duct stones, remnant gall bladder stones and sphincter dysfunction [16].

Black and brown pigment gallstones are two kinds of stones which could be found inside CBD, but are morphologically, compositionally, and clinically distinct. Black stones form primarily in the gallbladder in sterile bile. The pathogenesis of black stones is probably related to nonbacterial, nonenzymatic hydrolysis of bilirubin conjugates. Conditions that create excessive unconjugated bilirubin, such as chronic hemolysis in hemoglobinopathies, cirrhosis, ineffective erythropoiesis, and ileal diseases, predispose a patient to the formation of black pigment stones [17, 18]. Brown stones form not only within the gallbladder but also within the intrahepatic and extrahepatic ducts; they are uniformly infected with enteric bacteria and are usually associated with ascending cholangitis. Brown stones are related to juxtapapillary duodenal diverticula and are the predominant type of de novo common bile duct stones. Cholecystectomy usually dramatically decreases of the risk of black pigment stone disease, whereas stones often recur after cholecystectomy for brown stone disease [18].

In our study, we found a similar result with most patients having brown pigment stones (69.4%), and cases with juxtapapillary diverticula were prone to have brown pigment stone, although the difference was not significant, which might be due to small case numbers. However, our results revealed some inconsistent findings of note. First, the scores of the CT Severity Index and severity of laboratory cholestasis were increased in the black pigment stone group. Second, there was clear evidence of infection, including C-reactive protein, cholangitis, and positive blood culture, in patients with black pigment stones. Third, the mortality rate was higher in the black pigment stone group, although non-significantly.

The properties of the stones may explain these findings, as black pigment stone has a hard consistency, and tends not to spontaneously migrate through ampulla of vater. A more important factor may be duration of stone impaction: black pigment stone originating from remnant gall bladder stones causes a longer period of CBD obstruction, leading to more severe pancreatitis and poor prognosis. Thus, for cholecystectomized patients with acute biliary pancreatitis, especially with black pigment stone, decompression of CBD by ERCP or surgery should not be delayed.

There were some limitations in our study: First, the difference between the two groups was decided by the pattern of stone, and no further analysis of stone material was done. Second, some of our cases received treatment for infections using antibiotics, which might have influenced the final outcomes. Third, major limitations of this study included the retrospective study design and unmeasured differences between the two groups.

In conclusion, most cases with prior cholecystectomy and acute biliary pancreatitis were prone to have brown pigment stone. Cholecystectomized patients with acute biliary pancreatitis due to black pigment stone had elevated laboratory cholestasis values, as well as higher rates of cholangitis.
